# Online monitoring of dissolved oxygen tension in microtiter plates based on infrared fluorescent oxygen-sensitive nanoparticles

**DOI:** 10.1186/s12934-015-0347-9

**Published:** 2015-10-09

**Authors:** Tobias Ladner, David Flitsch, Tino Schlepütz, Jochen Büchs

**Affiliations:** AVT, Aachener Verfahrenstechnik, Biochemical Engineering, RWTH Aachen University, Worringerweg 1, 52074 Aachen, Germany

**Keywords:** High-throughput screening, Online monitoring, Dissolved oxygen tension, Oxygen-sensitive nanoparticles, Optical measurement, Microtiter plate, Fluorescence tags

## Abstract

**Background:**

During the past years, new high-throughput screening systems with capabilities of online monitoring turned out to be powerful tools for the characterization of microbial cell cultures. These systems are often easy to use, offer economic advantages compared to larger systems and allow to determine many important process parameters within short time. Fluorescent protein tags tremendously simplified the tracking and observation of cellular activity in vivo. Unfortunately, interferences between established fluorescence based dissolved oxygen tension (DOT) measurement techniques and fluorescence-based protein tags appeared. Therefore, the applicability of new oxygen-sensitive nanoparticles operated within the more suitable infrared wavelength region are introduced and validated for DOT measurement.

**Results:**

The biocompatibility of the used dispersed oxygen-sensitive nanoparticles was proven via RAMOS cultivations for *Hansenula polymorpha, Gluconobacter oxydans*, and *Escherichia coli*. The applicability of the introduced DOT measurement technique for online monitoring of cultivations was demonstrated and successfully validated. The nanoparticles showed no disturbing effect on the online measurement of the fluorescence intensities of the proteins GFP, mCherry and YFP measured by a BioLector prototype. Additionally, the DOT measurement was not influenced by changing concentrations of these proteins. The k_L_a values for the applied cultivation conditions were successfully determined based on the measured DOT.

**Conclusions:**

The introduced technique appeared to be practically as well as economically advantageous for DOT online measuring in microtiter plates. The disadvantage of limited availability of microtiter plates with immobilized sensor spots (optodes) does not apply for this introduced technique. Due to the infrared wavelength range, used for the DOT measurement, no interferences with biogenic fluorescence or with expressed fluorescent proteins (e.g. YFP, GFP or mCherry) occur.

## Background

In the recent past, high-throughput screening systems such as the BioLector technology [1, 2] which is based on shaken microtiter plates (MTP), the µ24 system [3] as combination of bubble column and shaken bioreactor system or the stirred ambr bioreactors [4, 5] became true alternatives to shake flasks for microbial cultivations. Due to the increasing miniaturization, parallelization and automation more experiments can be cost efficiently conducted within a short time [6–9]. To augment the process knowledge already in early process development, it is of general interest to develop new tools and improve existing technologies for small-scale bioreactors [10].

**Fig. 1:**
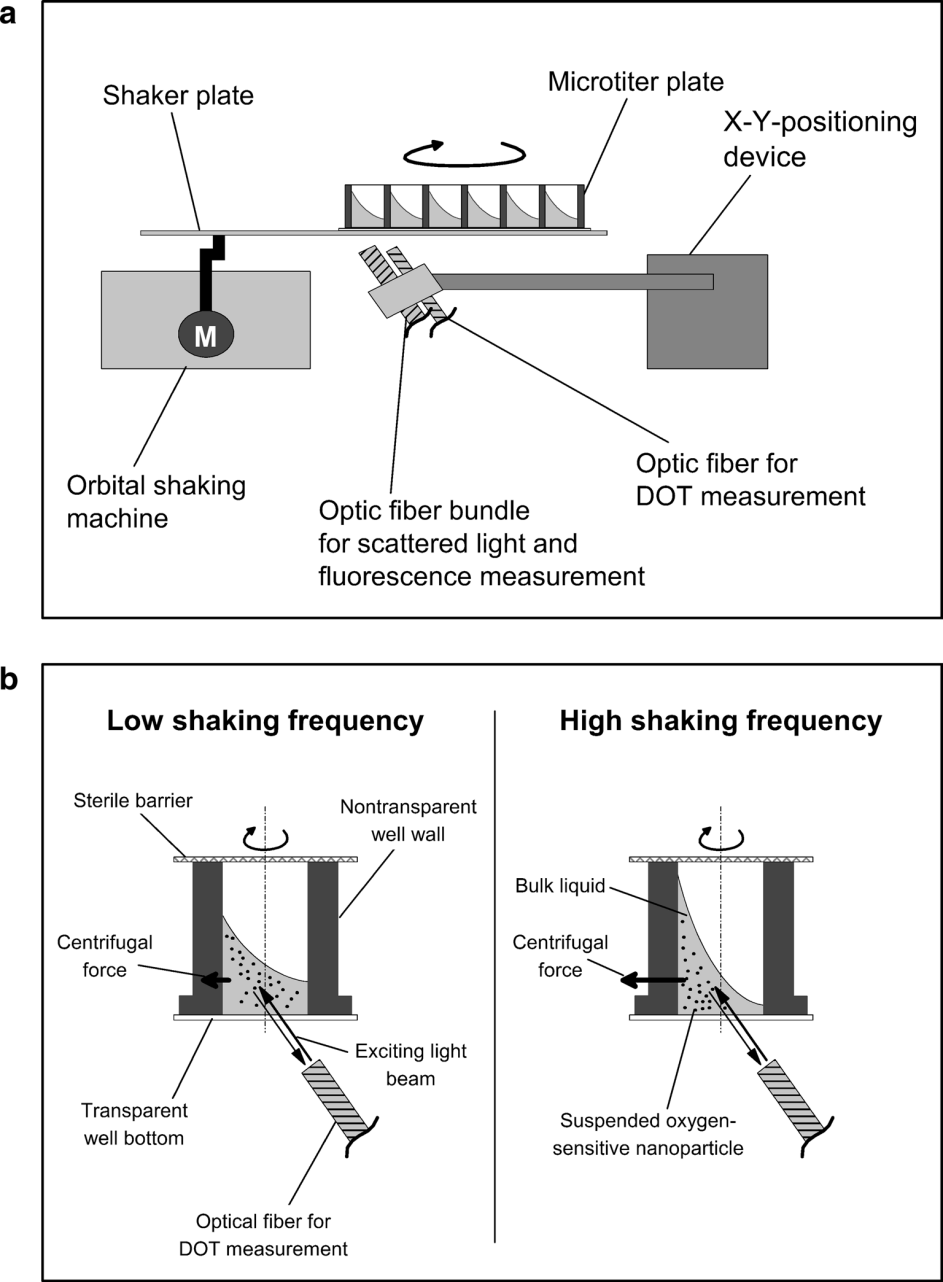
BioLector device. **a** Measurement setup of the BioLector device equipped with an optic fiber for DOT measurement and an optic fiber bundle for fluorescence measurement. **b** Influence of the centrifugal force acting on the bulk liquid and the dispersed oxygen-sensitive nanoparticles

The BioLector system has been introduced and proven as powerful technology for quasi-continuous online monitoring of cultivations in MTPs. With the help of an optical fiber bundle and a fluorescence spectrometer the online measurement of scattered light as online biomass signal and biogenic fluorescences was made accessible (Fig. 1a) [1, 2]. Huber et al. [11] have combined the BioLector technology with a liquid handling system (RoboLector) to reduce the experimental efforts and increase the reproducibility. The Bio- and RoboLector platform have become widespread during the last years and is used in numerous biotechnological applications [2, 12–15]. Besides the scattered light and biogenic fluorescences the dissolved oxygen tension (DOT) of the cultivation broth is another important process parameter. The DOT becomes accessible in MTPs and in small scale stirred bioreactor systems via oxygen-sensitive fluorescence dyes [16, 17]. These dyes change their fluorescence behavior in response to DOT and are usually immobilized as sensor spots (optodes) at the bottom of the cultivation vessel.

Kunze et al. [18] called attention to potential pitfalls of optical measurement systems for online monitoring of microbial cultivations. Optical DOT and pH measurements can be strongly affected by the expression of certain fluorescence proteins. Particularly, the expression of yellow fluorescent protein (YFP) and mCherry led to unreasonable DOT and pH measurements. The overlapping excitation wavelengths of the expressed fluorescent proteins and the oxygen sensitive fluorescent dyes for the DOT measurement (505 nm) were identified as reason for the interferences [18]. Borisov et al. [19, 20] introduced a new fluorescence-based optical oxygen sensor with red light excitation that should be more suitable to avoid overlapping absorption ranges of the sensor dye and biogenic fluorescing compounds. The oxygen-sensitive fluorescence dye (cyclometallated iridium(III) complex) was immobilized in nanoparticles which consist of a hydrophobic polystyrene core and a hydrophilic poly (vinylpyrrolidone) shell [20]. Because of the hydrophilic shell the oxygen-sensitive nanoparticles remain well dispersed in aqueous environments.

The aim of this study was to investigate the applicability and suitability of dispersed nanoparticles containing an oxygen-sensitive fluorescence dye with red light excitation for DOT measurement within the BioLector system. Impacts of the dispersed oxygen-sensitive nanoparticles on the growth behavior of *Gluconobacter oxydans*, *Hansenula polymorpha* and *Escherichia coli* were examined in shake flask cultivations with respiration activity monitoring. With the help of the obtained DOT values the respective k_L_a values of the applied cultivation conditions inside the used MTP could be determined.

## Results and discussion

### Biocompatibility of the dispersed oxygen-sensitive nanoparticles

**Fig. 2:**
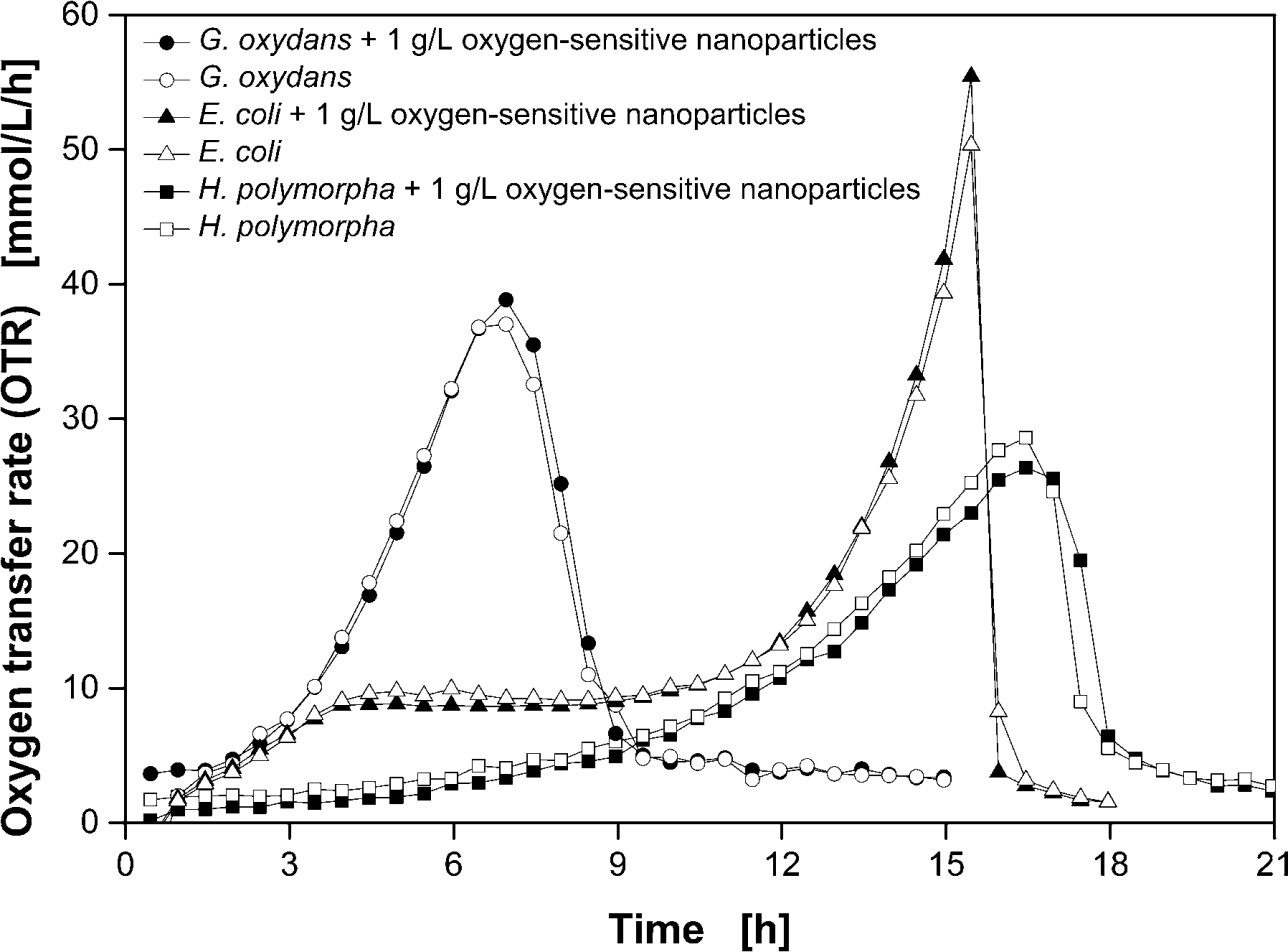
Biocompatibility of dispersed oxygen-sensitive nanoparticles. *G.* *oxydans* Δupp, *E.* *coli* BL21 (De3) pRSet-mCherry and *H.* *polymorpha* RB11 (P_FMD_-GFP) were cultivated in the RAMOS device to determine the oxygen transfer rates (OTR) with and without 1 g L^−1^ dispersed oxygen-sensitive nanoparticles. Cultivation conditions: *G.* *oxydans*: complex mannitol medium, 30 °C; *E.* *coli*: synthetic Wilms-MOPS auto-induction medium with 0.55 g L^−1^ glucose, 2 g L^−1^ lactose and 5 g L^−1^ glycerol, 37 °C; *H.* *polymorpha*: synthetic Syn-6-MES medium with 10 g L^−1^ glycerol, 30 °C; Shaking conditions: V_L_ = 10 mL in 250 mL RAMOS shake flask, n = 350 rpm, d_0_ = 50 mm

In 2013, Meier et al. introduced an easy and sensitive analytical method to investigate the biocompatibility of polymer materials based on the respiration activity [21]. The monitoring of the respiration activity is made possible by the RAMOS technology [22, 23] (HiTec Zang GmbH, Herzogenrath, Germany and Adolf Kühner AG, Birsfelden, Switzerland). In Fig. 2 the results of the corresponding biocompatibility tests for the dispersed oxygen-sensitive nanoparticles used in this work for DOT measurement are shown. The growth of *G. oxydans*, *E. coli* and *H. polymorpha* was monitored with and without 1 g L^−1^ dispersed nanoparticles. The OTR curves for both approaches of all three organisms are coinciding during the whole cultivations and no differences of the respiration activities became visible. Thus, the biocompatibility of the dispersed oxygen-sensitive nanoparticles for the investigated microorganisms has been proved. A discussion of each microorganism’s growth behavior is carried out in the following sections.

### Characterization of dispersed oxygen-sensitive nanoparticles under cultivation conditions

**Fig. 3:**
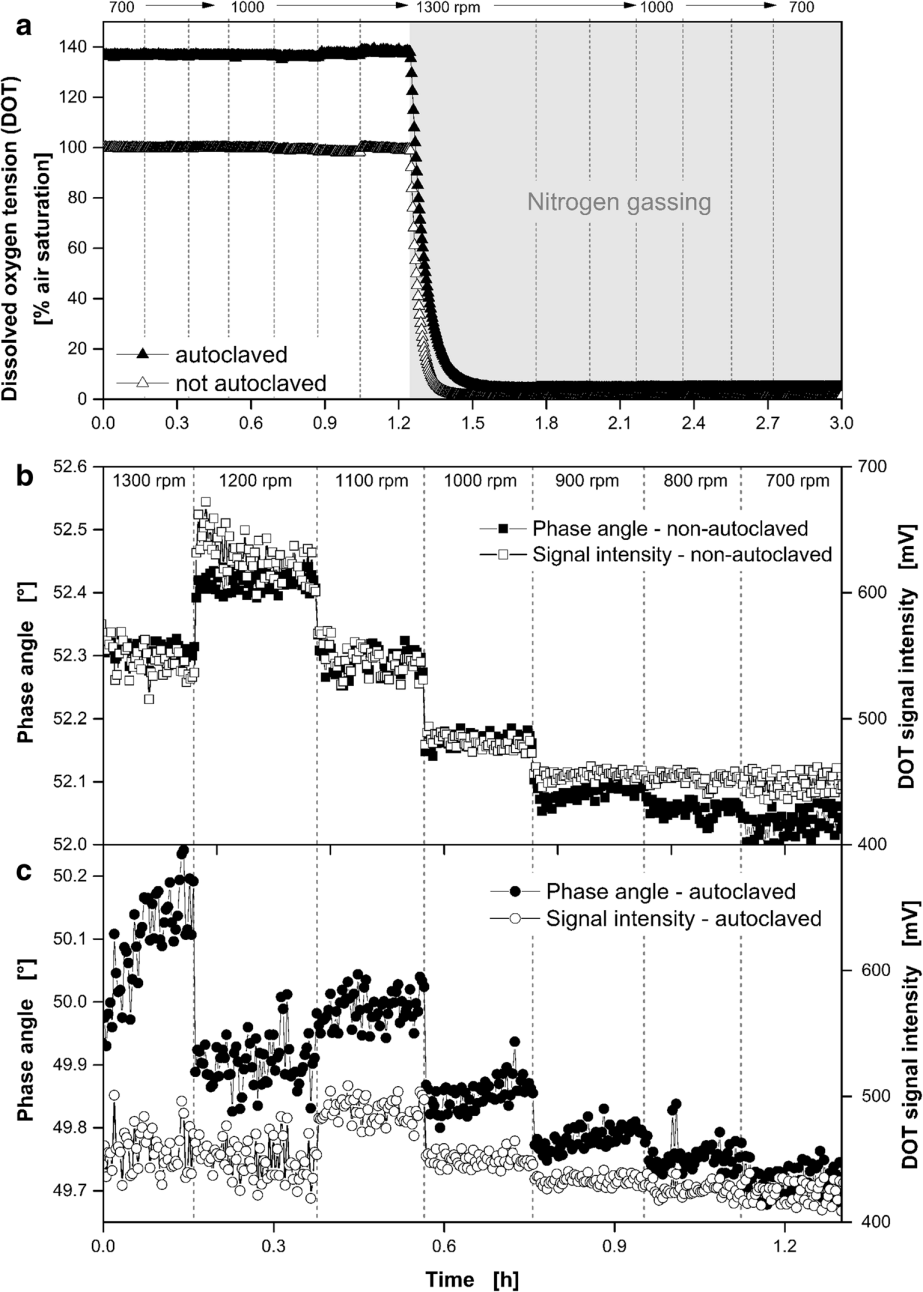
Influence of autoclaving and the impact of the shaking frequency on the DOT measured inside a microtiter plate via dispersed oxygen-sensitive nanoparticles. Influence of autoclaving (121 °C, 21 min) (**a**). Impact of the shaking frequency on the signal intensity and on the phase angle as raw signal for the DOT measurement via non-autoclaved (**b**) and autoclaved dispersed oxygen-sensitive nanoparticles (**c**). Measuring conditions: synthetic Wilms-MOPS medium without glucose and with 1 g L^−1^ dispersed oxygen-sensitive nanoparticles, 48well round well MTP without optodes, V_L_ = 800 µL, d_0_ = 3 mm, 30 °C; n varies and is given in the *figure*

The influence of autoclaving and the impact of the shaking frequency on the DOT measured inside a MTP via dispersed oxygen-sensitive nanoparticles are illustrated in Fig. 3. Prior to the experiment a one point calibration (100 % air saturation) was performed with non-autoclaved nanoparticles dispersed in Wilms-MOPS medium without glucose (Fig. 3a). Under gassing with ambient air the DOT values of the autoclaved dispersion is shifted from 100 % to roughly 140 % air saturation. Likewise, in the second (nitrogen gassing) phase of this experiment the measured DOT values of the autoclaved nanoparticles were shifted upwards from 0 % to approximately 4 % air saturation compared to the DOT values of the non-autoclaved dispersed nanoparticles. The reason for this DOT shift is shown in Fig. 3b. The measured phase angle as raw signal for the DOT determination was shifted to slightly smaller phase angles after autoclaving for all shaking frequencies (Fig. 3c). This result indicates that the fluorescence lifetime slightly changed due to the autoclaving process. Nevertheless, the nanoparticles still remained sensitive to dissolved oxygen, but a new two point calibration is required for correct DOT measurement.

The DOT signal intensity of the measurement with non-autoclaved and autoclaved dispersed oxygen sensitive nanoparticles is shown in Fig. 3b, c, respectively. Strikingly, the measured phase angle and DOT signal intensity are correlated. With an increased DOT signal intensity also the phase angle increases. Steps caused by changes of the shaking frequency were obvious in the phase angle as well as intensity signals. These steps might be attributed to the different centrifugal force acting on the rotating bulk liquid and on the particles with varying shaking frequency. Thus, different amounts of nanoparticles might be located within the optical detection volume of the oxygen sensor (compare Fig. 1b, c). Surprisingly, there is no steady increase with increasing shaking frequency observable over the whole frequency range. In the case of the non-autoclaved dispersed nanoparticles a maximal phase angle of approximate 52.4° was detected at a shaking frequency of 1200 rpm. After autoclaving the corresponding value shifted to roughly 50.1° at a shaking frequency of 1300 rpm. To compensate the influence of the shaking frequency or autoclaving on the measurement, it is necessary to perform the calibration and cultivation under equal conditions. The magnitude of fluorescence bleaching due to autoclaving of the dispersed nanoparticles can be determined by comparing the DOT signal intensities before and after autoclaving (Fig. 3b, c). In the shaking frequency range from 700 to 900 rpm, were the DOT signal intensity is not effected by the shaking frequency, the corresponding DOT signal intensities are roughly 450 mV before and 420 mV after autoclaving. This determines the loss of DOT signal intensity to roughly 7 % due to autoclaving. This might be attributed to a cluster formation of the dispersed nanoparticles or a possible molecular alteration due to the applied elevated temperature and pressure during autoclaving.

**Fig. 4:**
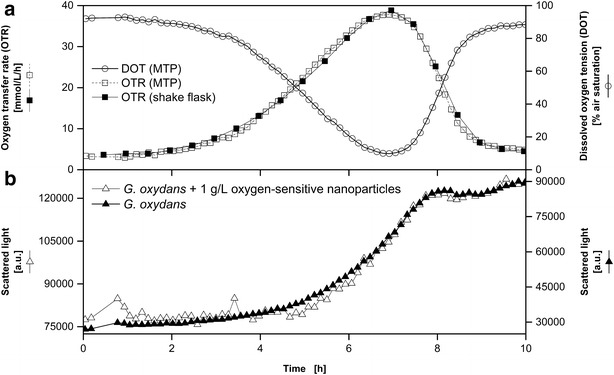
Comparison of *G.* *oxydans* Δupp cultivations using the BioLector microtiter plate and the RAMOS shake flask system. Online monitoring of the oxygen transfer rate (OTR) in a RAMOS shake flask and of the dissolved oxygen tension (DOT) measured via dispersed oxygen-sensitive nanoparticles in a MTP (**a**). *Scattered light curves* of cultivations with and without dispersed oxygen-sensitive nanoparticles in a MTP in the BioLector system (**b**). Cultivation conditions: BioLector: 48well Round Well Plate without optodes, V_L_ = 800 µL, n = 1000 rpm, d_0_ = 3 mm, 30 °C; RAMOS: 250 mL-RAMOS shake flask, V_L_ = 10 mL, n = 350 rpm, d_0_ = 50 mm, 30 °C; complex mannitol medium. Based on the measured DOT in MTP the OTR was calculated for the MTP with a fitted k_L_a-value of 186 h^−1^ according to Eq. 2. For a better comparison of the propagation of the *scattered light* signals, two specifically adjusted y axes were used

**Fig. 5:**
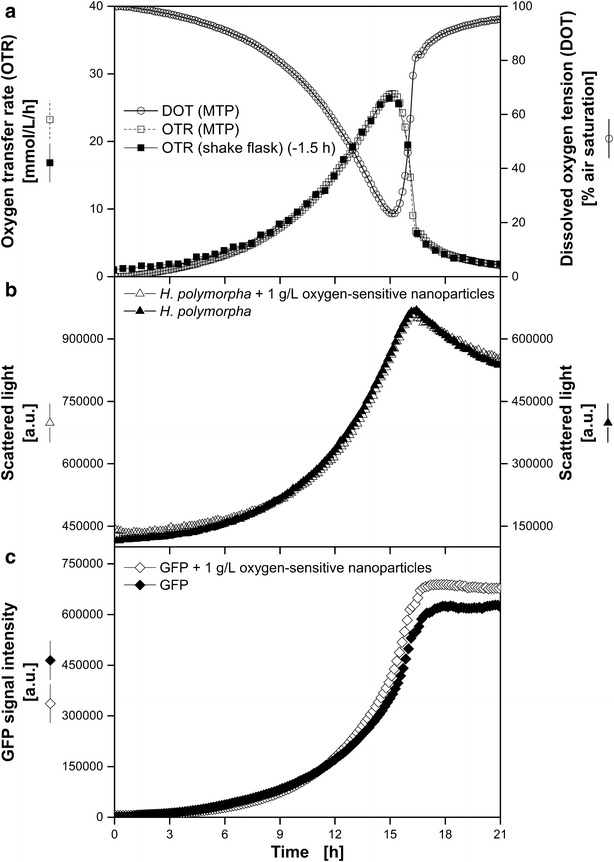
Comparison of *H.* *polymorpha* RB11 P_FMD_-GFP cultivations using the BioLector microtiter plate and the RAMOS shake flask system. Online monitoring of the oxygen transfer rate (OTR) in a RAMOS shake flask and dissolved oxygen tension (DOT) via dispersed oxygen-sensitive nanoparticles in a MTP (**a**) and microbial growth via scattered light in a microtiter plate in the BioLector system (**b**). **c** Fluorescence intensity of GFP (ex.: 490 nm/em.: 510 nm). Cultivation conditions: BioLector: 48well Round Well Plate without optodes, V_L_ = 800 µL, n = 1000 rpm, d_0_ = 3 mm, 30 °C; RAMOS: 250 mL-RAMOS shake flask, V_L_ = 10 mL, n = 350 rpm, d_0_ = 50 mm, 30 °C; synthetic Syn-6-MES medium with 10 g L^−1^ glycerol. Based on the DOT measured in MTP the OTR was calculated with a fitted k_L_a-value of 188 h^−1^ according to Eq. 2. The data of the RAMOS cultivation was shifted –1.5 h. For a better comparison of the propagation of the scattered light signals, two specifically adjusted y axes were used

**Fig. 6:**
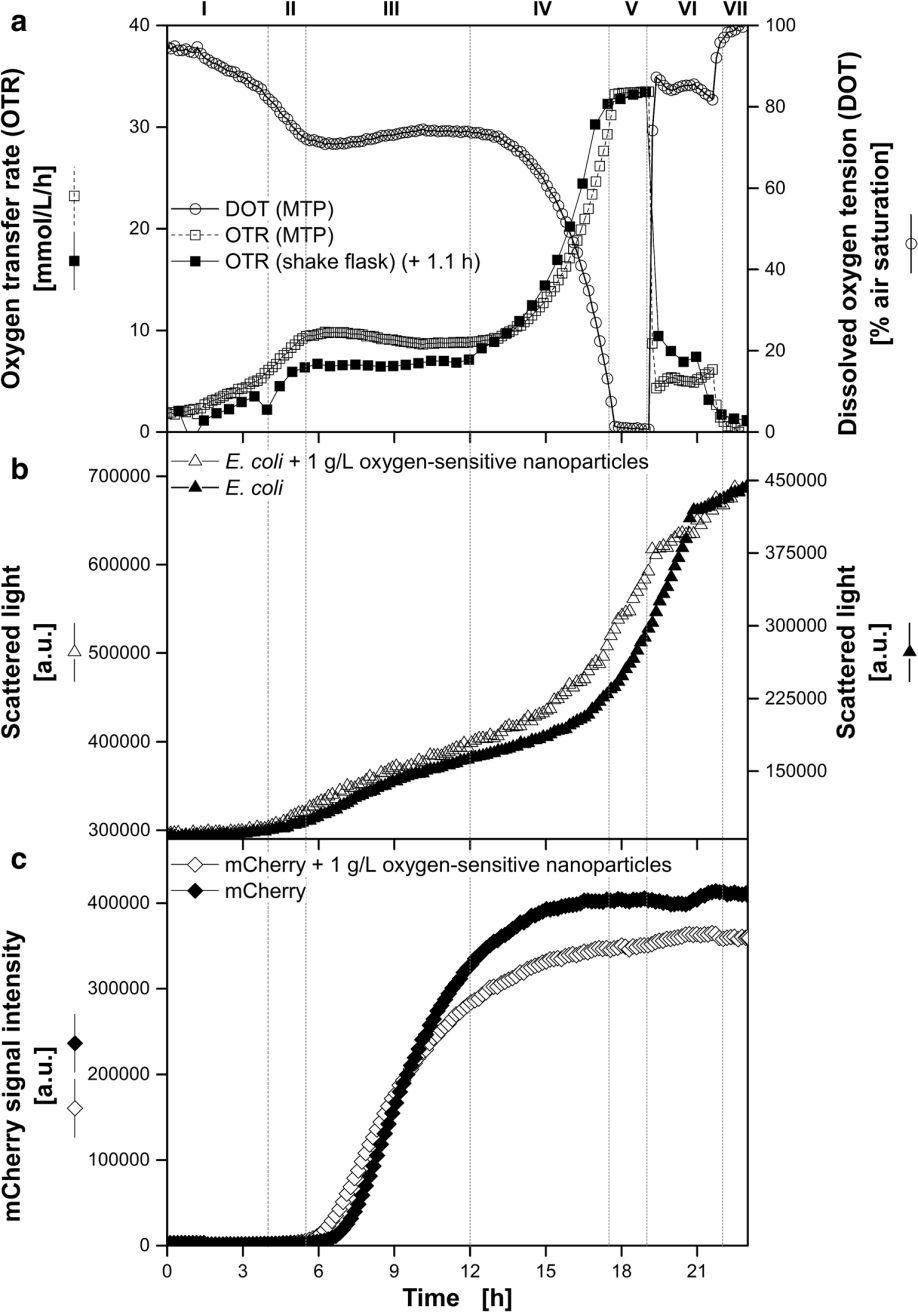
Comparison of *E.* *coli* BL21 (DE3) pRSet-mCherry cultivations using the BioLector microtiter plate and the RAMOS shake flask system. Online monitoring of the oxygen transfer rate (OTR) in a RAMOS shake flask and dissolved oxygen tension (DOT) via dispersed oxygen-sensitive nanoparticles in a MTP (**a**) and microbial growth via scattered light in a microtiter plate in the BioLector system (**b**). **c** Fluorescence intensity of mCherry (ex.: 580 nm/em.: 610 nm). Cultivation medium: Synthetic Wilms-MOPS auto-induction medium with 0.55 g L^−1^ glucose, 2 g L^−1^ lactose and 5 g L^−1^ glycerol. Cultivation conditions: BioLector: 48well Round Well Plate without optodes, V_L_ = 900 µL, n = 1000 rpm, d_0_ = 3 mm, 37 °C; RAMOS: 250 mL RAMOS shake flask, V_L_ = 23 mL, n = 350 rpm, d_0_ = 50 mm, 37 °C. Based on the DOT measured the OTR was calculated with a fitted k_L_a-value of 181 h^−1^ according to Eq. 2. The data of the RAMOS cultivation is shifted 1.1 h. For a better comparison of the propagation of the scattered light signals, two specifically adjusted y axes were used

**Fig. 7:**
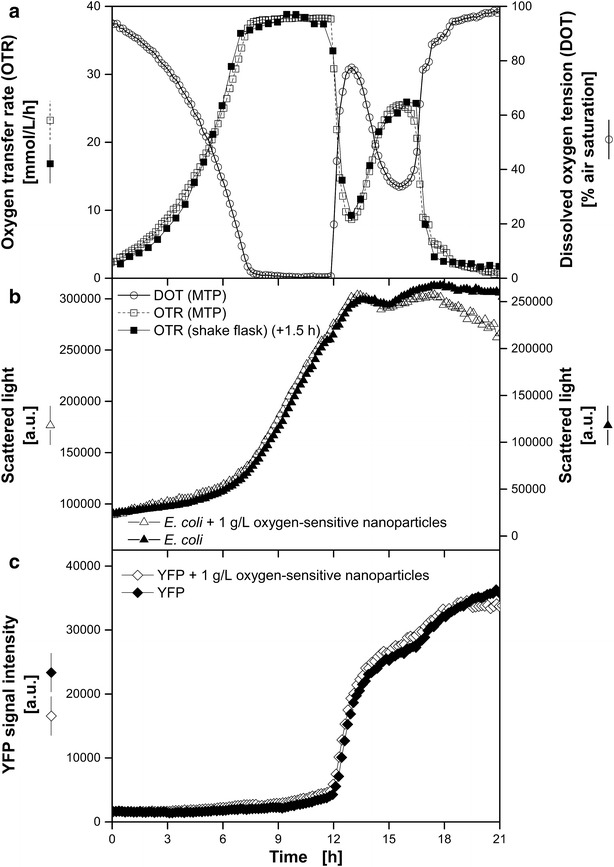
Comparison of *E.* *coli* BL21 (DE3) pRotHi-YFP cultivations using the BioLector microtiter plate and the RAMOS shake flask. Online monitoring of the oxygen transfer rate (OTR) in a RAMOS shake flask and dissolved oxygen tension (DOT) via dispersed oxygen-sensitive nanoparticles in a MTP (**a**) and microbial growth via scattered light in a microtiter plate in the BioLector system (**b**). **c** Fluorescence intensity of YFP (ex.: 510 nm/em.: 532 nm). Cultivation medium: Synthetic Wilms-MOPS medium with 20 g L^−1^ glucose and 1.5 g L^−1^ sorbitol. Cultivation conditions: BioLector: 48well Round Well Plate without optodes, V_L_ = 800 µL, n = 1000 rpm, d_0_ = 3 mm, 37 °C; RAMOS: 250 mL RAMOS shake flask, V_L_ = 22 mL, n = 350 rpm, d_0_ = 50 mm, 37 °C. Based on the DOT measured in MTP the OTR was calculated with a fitted k_L_a-value of 211 h^−1^ according to Eq. 2. The data of the RAMOS cultivation is shifted 1.5 h. For a better comparison of the propagation of the scattered light signals, two specifically adjusted y axes were used

In the following Figs. 4, 5, 6, 7 DOT, scattered light representing biomass and fluorescence intensities of fluorescent tags (except Fig. 4), measured in MTPs, are presented. The OTR in shake flasks of equivalent reference cultures was quantified by measurements in a RAMOS device. In addition, the OTR was calculated for the MTP based on the measured DOT and OTR using Eq. 2 by fitting the k_L_a value. This approach, already introduced by Wewetzer et al. [24], is only justified, if equivalent culture conditions in shake flask and MTP can be assured. Due to the presence of nanoparticles light scattering increased and resulted in a signal offset compared to the measurement without dispersed nanoparticles. Hence, separate y-axes with different scales were chosen for the comparison of the scattered light signal in cultivations with and without dispersed oxygen sensitive nanoparticles.

The oxygen-sensitive nanoparticles can easily be removed together with the biomass by centrifugation or sterile filtration (pore size: 0.2 µm). However, since the main objective for the use of the oxygen-sensitive nanoparticles is upstream bioprocess development and screening on small scale, reuse of the oxygen-sensitive nanoparticles was not further investigated.

### Oxygen-unlimited cultivation of *G.* *oxydans* Δupp

In Fig. 4 cultivations of *G.* *oxydans* Δupp in unbuffered complex mannitol medium with and without dispersed oxygen-sensitive nanoparticles are compared with each other. Since the DOT values were above 0 % and no maximal oxygen transfer capacity plateau [22, 23] could be observed in the OTR curves, no oxygen-limitation occurred during the whole cultivations. The minimum of the DOT of 9.8 % air saturation in the MTP as well as the maximum of the OTR of 38.9 mmol L^−1^ h^−1^ in the shake flask were reached exactly at the same time after 7 h. The rapid decrease of the DOT in the MTP and simultaneously the increase in OTR in the shake flask were caused by the oxygen consumption during exponential growth of *G.* *oxydans* Δupp and the mannitol oxidation to fructose [25]. To calculate the OTR in the MTP from the DOT the k_L_a value of the MTP is needed. According to the performed parameter estimation of the k_L_a value the best approximation between OTR in MTP and shake flask was obtained with a k_L_a value of 186 h^−1^. With that k_L_a value the calculated OTR curve of the MTP perfectly coincides with the OTR curve of the shake flask during the whole cultivation (Fig. 4a). Likewise, the trends of the scattered light curves of the cultivations in the MTP with and without dispersed oxygen sensitive nanoparticles are comparable to the propagation of the OTR curves (Fig. 4b). Until the end of the exponential growth (7 h) the scattered light intensity increased exponentially. Subsequently, the bacteria entered into the stationary phase at about 8 h which was indicated by the scattered light signal levelling off on a plateau till the end of the experiment. In agreement with that trend the DOT increases and the OTR decreases.

### Oxygen-unlimited cultivation of *H.* *polymorpha* RB11 P_FMD_-GFP

After the applicability of the new DOT measuring system was shown for complex media, the cultivation of a genetically modified yeast strain on synthetic medium was investigated. A green fluorescence protein (GFP) expressing strain of *H.* *polymorpha* was chosen to detect potential interferences between the fluorescence-based DOT measurement and the frequently used GFP.

Figure 5 shows the cultivation of *H.* *polymorpha* RB11 P_FMD_-GFP on buffered synthetic Syn-6-MES medium in MTP and shake flask. In both cultivation systems almost identical OTRs were determined. Due to the separate medium preparation and inoculation (volumes <5 µL had to be pipetted) no absolutely identical starting conditions could be adjusted in the MTP and shake flask. Thus, a slightly lower initial biomass concentration in the shake flask than in MTP led to a longer lag-phase. According to the parameter estimation the best approximation between OTR in MTP and shake flask was obtained with a k_L_a value of 188 h^−1^ and a time shift between the two cultivation vessels of -1.5 h (Fig. 5a). Like in the cultivation of *G.* *oxydans* Δupp, no oxygen limitation occurred during the cultivations. At 15 h the maximal OTR in shake flask cultivation of 26.4 mmol L^−1^ h^−1^ and minimal DOT of 23.4 % air saturation in the MTP cultivation were measured (Fig. 5a). From 15 to 17 h the DOT and OTR curves increased and decreased, respectively, indicating the end of the exponential growth due to glycerol depletion [26]. Within the same time range, the scattered light intensities (Fig. 5b) of both MTP cultivations with and without dispersed oxygen sensitive nanoparticles turned from the exponential increase into a nearly linear decrease. This decrease of the scattered light signal is known from preceding experiments and can be attributed to morphological changes of the microorganisms [14]. Due to the substrate glycerol and the applied FMD promoter GFP expression was linked to microbial growth [27]. Accordingly, the GFP fluorescence intensities increased exponentially until the substrate was depleted (17 h) and then remained constant (Fig. 5c). With respect to the measured process parameters, no significant influence of the dispersed oxygen-sensitive nanoparticles was determined. Vice versa, also the GFP fluorescence did not show any impact on the DOT measurement similar to the results of the different measurement system Kunze et al. [18] used.

### Oxygen-limited cultivation of *E.* *coli* BL21 (DE3) pRSet-mCherry in auto-induction medium

To further challenge the measurement system based on the new oxygen-sensitive nanoparticles, an *E.* *coli* strain expressing mCherry was investigated. Kunze et al. [18] spotlighted the disturbing interference of mCherry on DOT measurements with conventional optodes. Even their proposed compensation method was not successful in this particular case. Figure 6 shows the cultivations of *E.* *coli* BL21 (DE3) pRSet-mCherry in a MTP and shake flask. As in the example above, the curve for the shake flask needed to be shifted for 1.1 h due to slight volume variations during main culture preparation. In this experiment a k_L_a value of 181 h^−1^ was estimated for the MTP cultivation. The calculated OTR in MTP and measured OTR in the shake flask did not coincide as well as in the previous examples, but were still in good agreement. Based on the DOT and OTR results, seven characteristic phases could be distinguished in this fermentation on autoinduction media containing three different carbon sources (Fig. 6a). The phases I—IV have already been published by Rahmen et al. [28]. In this mentioned study no cultivation under oxygen-limited conditions were investigated. After 4 h the first carbon source glucose was depleted indicated by a drop of the OTR and a change of the slope in the DOT (I). Subsequently, growth on glycerol followed for roughly 1.5 h (II). From 5.5 h simultaneous consumption of glycerol (as energy source) and product formation (mCherry) on lactose occurred with a constant OTR of about 6.5 mmol L^−1^ h^−1^ (III). Due to depletion of lactose this phase stopped after 12 h. Within the next 7 h the residual glycerol was consumed. This period could be divided into two phases: non-oxygen limited growth (IV: 12–17.5 h) and oxygen limited growth (V: 17.5–19 h) indicated by a plateau of the OTR curve (33.3 mmol L^−1^ h^−1^) and a DOT close to zero. Losen et al. [29] investigated the effects of oxygen limitations during *E.* *coli* cultivations. As a consequence of the oxygen limitation an enhanced production of acetate was reported. Growth on acetate was observed after depletion of the primary substrate. Thus, the OTR level of about 9.4 mmol L^−1^ h^−1^ in Fig. 6a after 19.5 h could be attributed to the consumption of acetate (VI). In the last phase (VII: 22–23 h) all carbon sources were depleted and the cultivation terminated. The different phases are also visible in the scattered light intensities (Fig. 6b). Analog to Kunze et al. [18], during the expression of mCherry a linear increase was detected (III) that turned into an exponential one when residual glycerol was consumed (IV + V). In Fig. 6c the fluorescence intensities of mCherry are depicted. In contrast to Kunze et al. [18], no significant influence of the dispersed oxygen-sensitive nanoparticles on the fluorescence measurement of mCherry was observed. Within phase I and II the fluorescence intensities remained close to zero. In both experiments, after 6 h the intensities started to increase due to mCherry expression, but diverged over the time. Most likely, the deviation of the presented cultivations can be attributed to slightly different starting conditions due to the separate media preparation.

### Oxygen-limited diauxic growth of *E.* *coli* BL21 (DE3) pRotHi-YFP

Kunze et al. [18] reported a moderate influences of yellow fluorescence proteins (YFP) on the DOT measurement and minimized these effect by a mathematical correction procedure. Thus, as last example the cultivation of *E.* *coli* BL21 (DE3) pRotHi-YFP on synthetic medium with two initial carbon sources was investigated (Fig. 7). Due to the DOT-based OTR calculation for the microtiter plate a time shift of +1.5 h of the flask cultivations and a k_L_a value of 211 h^−1^ was determined. Hansen et al. [30] presented a modified evaluation method for RAMOS measurements and demonstrated the advantages of this method with almost the same experiment. They divided the cultivation into three phases: consumption of glucose, consumption of sorbitol and consumption of acetate. All phases were clearly indicated by three individual peaks in the OTR curve. However, in Fig. 7a only two distinct phases can be identified. After 7 h an oxygen limitation occurred that lasted 4.5 h, indicated by a plateau of the OTR curve at 38 mmol L^−1^ h^−1^ and a constant DOT of 0 % air saturation. Within the second part (13–21 h) another OTR peak (26 mmol L^−1^ h^−1^) emerged. A separation of the growth phase on glucose and sorbitol are not visible in the DOT and OTR, due to the oxygen limitation. Subsequently, the second peak can be referred to metabolic activity on acetate. Since this study focuses on the technical validation of the new measurement system no further investigations were conducted to explain the growth kinetics. For the purpose of technical validation, the identical pattern of the OTR measured in shake flask and the DOT-based OTR in MTP were sufficient. In Fig. 7b the scattered light intensities are plotted. After 4 h the signal increased exponentially and turned into a linear increase (7 h) during the oxygen limitation. With the end of the oxygen limitation (11.5 h) the scattered light intensities remained constant and increased again slightly due to growth on acetate (15–18 h). During the oxygen limited growth on glucose and sorbitol no significant increase of YFP fluorescence could be detected (Fig. 7b). In contrast, a sudden increase of the YFP fluorescence signal occurred due to YFP maturation at sufficiently high DOT. It is known that the limiting step of the YFP maturation is the oxidation of the chromophore [31]. Since almost all available oxygen was consumed for microbial growth during the oxygen limitation only small YFP maturation rates were observed during this growth phase. Analog to Kunze et al. [18] expressed and non-oxidized YFP molecules mature directly after the end of the oxygen limitation (12 h) and a sudden increase of the fluorescence signal was detected. No significant influence of the dispersed oxygen-sensitive nanoparticles on growth or YFP formation could be determined. In contrast to Kunze et al. [18], the YFP fluorescence did not show any impact on the DOT measurement.

### Potential improvements of the measuring method

**Fig. 8:**
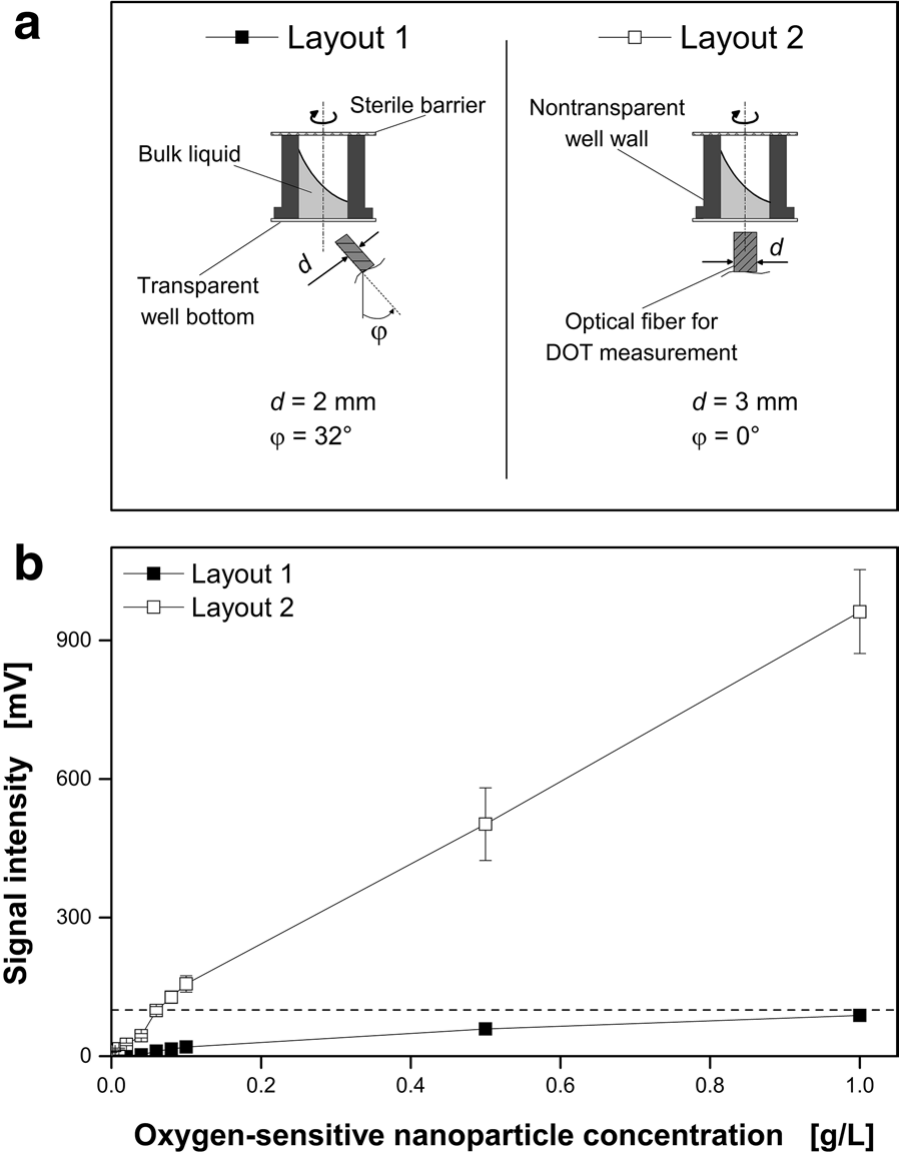
Dependency of the dispersed oxygen-sensitive nanoparticles concentration and the optical fiber orientation below the microtiter plate on the signal intensity. **a** Two different geometrical layouts below the MTP were tested with different orientation and optical fiber diameter. **b** The minimum signal intensity of 100 mV (*dashed line*) is required for a reliable DOT measurement. Measurement conditions: synthetic Wilms-MOPS medium without glucose, 48well Round Well Plate without optodes, V_L_ = 800 µL, n = 1000 rpm, d_0_ = 3 mm, 23 °C

In the present study, a recommended oxygen-sensitive nanoparticle concentration of 1 g L^−1^ was used for all cultivations to obtain a minimum DOT signal intensity of 100 mV [32]. With respect to the relative high cost of these particles (580 € per 50 mg; 03/2015), investigations were conducted to reduce the standard concentration while still allowing reliable measurements in MTPs. In Fig. 8a the standard and an improved setup for the DOT measurement below the MTP are presented. By using Layout B, the required minimal signal intensity (dashed line) is already reached with a dispersed oxygen-sensitive nanoparticles concentration of 0.06 g L^−1^. Thus, due to a change of the angle of the optical fiber to the vertical well center from 32° to 0° and by replacing the optical fiber (diameter: 2 mm) with an optical fiber rod (3 mm) significant improvements were achieved. For further applications the costs per cultivation can markedly be reduced by using Layout B due to the decreased minimum required nanoparticles concentration.

While the costs for 48 cultivations (volume per well: 1 mL, nanoparticles concentration: 1 g L^−1^) in a MTP would amount to 556.80 € with Layout A, only 33.41 € (volume per well: 1 mL, nanoparticles concentration: 0.06 g L^−1^) need to be invested for the same 48 experiments using Layout B. Compared to other commercially available MTPs equipped with dissolved oxygen optodes, the presented optical infrared DOT measurement via dispersed oxygen sensitive nanoparticles by using Layout B turned out to be a commercially reasonable alternative as single use disposable measurement system.

## Conclusion

Online and non-invasive monitoring systems for small-scale cultivations often rely on optical measurements to determine important process parameters. Kunze et al. [18] pointed at potential pitfalls of optical DOT measurements in the presence of fluorescence proteins. Within this study, a new online optical infrared DOT monitoring system via dispersed oxygen-sensitive nanoparticles has been presented. The biocompatibility of the utilized dispersed oxygen-sensitive nanoparticles was proven for *H.* *polymorpha*, *G.* *oxydans* and *E.* *coli*. Furthermore, the importance of system calibration under cultivation conditions was demonstrated. Due to the red light excitation no interferences with biogenic fluorescences were observed. In contrast to other DOT measurement methods [18], also GFP, mCherry and YFP had no impact on the DOT measurement. K_L_a values for the applied cultivation conditions were estimated to calculate the OTR in MTP based on the DOT. OTRs calculated for the MTPs and OTRs determined in shake flasks via the RAMOS technology agreed very well with each other. No influences on the growth kinetics and oxygen supply caused by the dispersed nanoparticles were detected.

Since optodes at the well bottom are no longer needed, the measurement system can be applied for various MTPs. Finally constraints due to rather expensive MTPs with immobilized sensor spots (optodes) can be overcome with this introduced technology. Furthermore, the ability of the dispersed oxygen-sensitive nanoparticles to withstand autoclaving is an additional advantage for sterile cultivations. A new layout for the measurement setup was proposed, to reduce the minimal required concentration of dispersed oxygen-sensitive nanoparticles. Thus, the costs per cultivation could significantly be reduced and the system provides a serious alternative to established devices.

## Methods

### Microorganisms

In this study four different microorganisms were applied: *Gluconobacter oxydans* 621 H ∆upp, *Hansenula polymorpha* RB11 pC10-FMD P_FMD_-GFP expressing the green fluorescent protein GFP, *Escherichia coli* BL21 (DE3) pRSet mCherry expressing the red fluorescent protein mCherry and having an ampicillin resistance, as well as *Escherichia coli* BL21 (DE3) pRotHi YFP expressing the yellow fluorescent protein YFP and having a kanamycin resistance. These strains were cultivated as follows in complex mannitol medium, synthetic Syn-6-MES medium, synthetic Wilms-MOPS medium containing glucose, lactose and glycerol, as well as synthetic Wilms-MOPS medium containing glucose and sorbitol, respectively.

### Media and cultivation

All pre-cultures were carried out in shake flasks. The cultivation vessels for the main cultures were 250 mL RAMOS shake flasks for the RAMOS measurements and MTPs for the DOT, scattered light and fluorescence measurements. Every main culture medium for DOT measurements contained 1 g L^−1^ dispersed oxygen–sensitive nanoparticles if not otherwise specified. All main cultivations were inoculated with pre-cultures adjusting a starting OD_600_ of 0.1. If not otherwise mentioned, all media were autoclaved for 20 min at 121 °C and 1 bar for sterilization.

The complex mannitol medium for the pre- and main cultivation of *G.* *oxydans* 621 H Δupp consisted of 5 g L^−1^ yeast extract, 20 g L^−1^ mannitol, 1 g L^−1^KH_2_PO_4_, 1 g L^−1^ (NH_4_)_2_SO_4_, 2.5 g L^−1^ MgS0_4_·7H_2_O and 50 mg L^−1^ cefoxitin dissolved in deionized water. All reagents were of analytical grade and purchased from Carl Roth GmbH & Co. (Karlsruhe, Germany). The pH was adjusted with 5 M KOH to a value of 6. The pre-culture was inoculated with a cryoculture containing additionally 200 g L^−1^ glycerol.

Synthetic Syn-6-MES medium for the pre- and main cultivation of *H.* *polymorpha* was prepared according to Jeude et al. [33]. The pre-cultivation was inoculated with a cryoculture (200 g L^−1^ glycerol stocks). For the main cultivation of *H.* *polymorpha* within the MTP 1 g L^−1^ dispersed oxygen–sensitive nanoparticles were added to the Syn-6-MES medium.

For the two *E.* *coli* clones two pre-cultivations were conducted. For the first pre-cultivation terrific broth (TB) medium consisting of 12 g L^−1^ tryptone, 24 g L^−1^ yeast extract, 12.54 g L^−1^ K_2_HPO_4_, 2.31 g L^−1^ KH_2_PO_4_, and 5 g L^−1^ glycerol (all ingredients from Roth, Germany) dissolved in water was used. The pH value was not adjusted and was 7.2 ± 0.2. The first pre-cultivations were inoculated with complex medium (TB) cryocultures (200 g L^−1^ glycerol stocks). Modified Wilms and Reuss medium (henceforth referred as Wilms-MOPS medium) [34] for the second pre-cultivation of the two *E.* *coli* clones consisted of 5 g L^−1^ (NH_4_)_2_SO_4_, 0.5 g L^−1^ NH_4_Cl, 3.0 g L^−1^ K_2_HPO_4_, 2 g L^−1^ Na_2_SO_4_, 0.5 g L^−1^ MgSO^4^·7H_2_O, 0.01 g L^−1^ thiamine hydrochloride, 20.9 g L^−1^ 3-(*N*-morpholino)-propanesulfonic acid (MOPS, 0.2 M), 20 g L^−1^ glucose and 1 mL L^−1^ trace element solution. The trace element solution contained 1.98 g L^−1^ CaCl_2_·2H_2_O, 0.54 g L^−1^ CoCl_2_·6H_2_O, 0.48 g L^−1^ CuSO_4_·5H_2_O, 41.76 g L^−1^ FeCl_3_·6H_2_O, 0.3 g L^−1^ MnSO_4_·H_2_O, 0.54 g L^−1^ ZnSO_4_·7H_2_O, 33.39 g L^−1^ Na_2_EDTA (Titriplex III). The pH was adjusted with 5 M NaOH to a value of 7. Dependent on the resistance of the clones 50 mg L^−1^ kanamycin or 100 mg L^−1^ ampicillin were added to the medium. For the main cultivation of *E.* *coli* BL21 (DE3) pRSet mCherry a modified Wilms-MOPS autoinduction medium was used. Compared to the medium of the second pre-culture, the 20 g L^−1^ glucose were replaced by 0.55 g L^−1^ glucose, 2 g L^−1^ lactose and 5 g L^−1^ glycerol. Additionally 1 g L^−1^ dispersed oxygen-sensitive nanoparticles were added to the basis solution of the Wilms-MOPS medium for the MTP cultivations and autoclaved. For the main cultivation of *E.* *coli* BL21 (DE3) pRotHi YFP the modified Wilms-MOPS medium of the second pre-cultivation was supplemented with 1.5 g L^−1^ sorbitol.

### Measurement of scattered light and fluorescence intensities and DOT in MTPs

The in-house constructed BioLector system was operated with a FluoroMax-4 spectrofluorometer (HORIBA Jobin Y, Munich, Germany) and enabled the measurement of scattered light and fluorescence intensities during the cultivations. It was equipped with a Y-shaped optical fiber (UV–VIS, LEONI Fiber Optics GmbH, Neuhaus-Schierschnitz, Germany). The applied wavelengths and spectrometer settings for the BioLector online monitoring are summarized in Table 1. The applied 48 deep-well MTP (MTP-R48-B, m2p-labs GmbH, Baesweiler, Germany) was sealed with an oxygen permeable foil (AeraSeal film, A9224-50EA, Sigma-Aldrich Chemie GmbH, Steinheim, Germany). With the help of an in-house constructed thermo chamber a constant cultivation temperature was ensured.

**Table 1:** Optical signals and applied setup for BioLector online monitoring

Optical signal	λ_ex_ (nm)	λ_em_ (nm)	Integration time (ms)	Slit (nm)
Biomass (scattered light)	650	–	900	6
DOT	620	760	128	–
GFP fluorescence	488	520	600	8
YFP fluorescence	510	532	600	8
mCherry fluorescence	580	610	600	8

For the DOT measurement a PICO2OEM sensor (Pyro Science GmbH, Aachen, Germany) with a PICFIB_2_ optical fiber (Pyro Science GmbH, Aachen, Germany) was integrated in the BioLector (Fig. 1a). Oxygen-sensitive nanoparticles (OXNANO, Pyro Science GmbH, Aachen, Germany) were dispersed within the respective medium to enable the DOT measurement (Fig. 1b). The concentration of 1 g L^−1^ was used to obtain the recommended signal intensity of 100 mV with the PICO2OEM + PICFIB_2_. The current cost of the oxygen-sensitive nanoparticles is 11.60 € mg^−1^ and a further cost analysis is accomplished in results and discussion.

### Fluorescence-based DOT calculations

The following modified Stern–Volmer equation (Eq. 1) was used to calculate the DOT based on the measured phase angle [32]:

1 $$\frac{{\tan { {{\Phi }} }}}{{\tan {{{\Phi }}}_{0} }} = \frac{f}{{1 + K_{SV} \cdot DOT}} + 1 - f$$

where *Φ* is the phase angle [°], *Φ*_*0*_ is the phase angle with DOT equaling zero [°], *f* is a specific parameter for the used measurement system (0.89, PICO2OEM) and K_SV_ is the Stern–Volmer constant [–]. To determine the phase angle in absence of oxygen (*Φ*_*0*_) the cultivation chamber was gassed with pure nitrogen. To determine the Stern–Volmer constant K_SV_ a second calibration point was obtained by gassing the cultivation chamber with ambient air (DOT = 100 %).

### DOT-based OTR calculation

Wewetzer et al. [24] demonstrated a method to calculate OTR values based on DOT measurements. In brief, the OTR can be calculated from the measured DOT according to the following equation:

2 $$OTR = k_{L} a \cdot L_{{O_{2} }} \cdot \left( {pO_{2}^{gas} - \frac{DOT}{100} \cdot pO_{2}^{cal} } \right)$$

While the oxygen solubilities ($L_{{O_{2} }}$) of the fermentation media were calculated according to literature [35–37], the volumetric oxygen transfer coefficients $(k_{L} a)$ were approximated by the method of least squares. $pO_{2}^{gas}$ represents the oxygen partial pressure in the headspace of the MTP and is assumed to be constant (0.21 bar) as well as the ambient oxygen partial pressure during calibration $pO_{2}^{cal}$ (0.21 bar). In Wewetzer et al. [24] the same “mastermix” (medium plus microorganisms) was used for both culture systems, the shake flask and MTP, to adjust identical starting conditions. This was not possible in the present study due to the required separate medium preparation with dispersed oxygen-sensitive nanoparticles. Therefore, slight inoculation variances resulted. To account for differently long lag-phases the algorithm to calculate the sum of squared errors was extended for the present study to consider a possible time shift. Thus, two parameters (time shift and k_L_a) were estimated for the conversion of DOT to OTR.

## Abbreviations

DOT: dissolved oxygen tension [% air saturation]; *E. coli* mCherry: *Escherichia coli* BL21 (De3) pRSet—mCherry; *E. coli* YFP: *Escherichia coli* BL21 (De3) pRotHi—YFP; *G.* *oxydans*: *Gluconobacter* *oxydans* Δupp; GFP: green fluorescent protein; *H.* *polymorpha*: *Hansenula* *polymorpha* RB11 PFMD-GFP; MTP: microtiter plate; OTR: oxygen transfer rate [mol L^−1^ h^−1^]; OD_600_: optical density at a wavelength of 600 nm; RAMOS: respiration activity monitoring system; YFP: yellow fluorescent protein.

### List of symbols

d_0_: shaking diameter [mm]; $f$: specific parameter for oxygen-sensitive nanoparticles and PICO2OEM (0.89) [–]; $\varPhi$: phase angle [°]; $\varPhi_{0}$: phase angle with DOT = 0 % air saturation [°]; $\varPhi_{100}$: phase angle with DOT = 100 % air saturation [°]; k_L_a: volumetric oxygen transfer coefficient [h^−1^]; K_SV_: Stern–Vollmer constant [–]; L_O2_: oxygen solubility [mol/l/bar]; λ_ex_, λ_em_: fluorescence excitation and emission wavelength [nm]; pO_2_^gas^: oxygen partial pressure in gas phase [bar]; pO_2_^gas^: oxygen partial pressure in gas phase during calibration [bar]; V_L_: liquid filling volume [mL].
